# Case report on the recurrence of tuberculosis of hip after 40 years

**DOI:** 10.1186/2193-1801-3-662

**Published:** 2014-11-06

**Authors:** Salim Jeddo, Chuan Wang Huang, Ming Li

**Affiliations:** Orthopedic Department, Qilu Hospital of Shandong University, 107 Wenhua West Street, Jinan, Shandong 250012 PR China; Department of Orthopedic, Qilu Hospital of Shandong University, Jinan, Shandong China

**Keywords:** Arthritic tuberculosis, Total hip arthroplasty, Anti-tuberculosis chemotherapy

## Abstract

Tuberculosis of joints is relatively rare condition and is associated with varied degree of immobility as well as other limitations. Tuberculosis of hip joint results in a remarkable decline in the living standard of the patients since hip joint has a wide range of function in daily movements apart from being the pivotal weight bearing joint in human body. The degeneration of hip joint culminates into long term morbidity for the patient. In this case report we present a patient who had suffered from the tuberculosis of hip joint 40 years before and had received the standard anti-tuberculosis chemotherapeutic regimen with isoniazid, rifampicin, pyrazinamide and ethambutol. After treatment, the patient had some degree of relief from the pain however the movement of the hip joint was restricted; the restriction increasing progressively since. On diagnostic testing he was found to have a recurrence of tuberculosis along with the old scar tissue left by the primary condition. The patient was assessed thoroughly after which total hip replacement surgery was performed along with the adhesiolysis. The patient made a remarkable recovery after the surgery with a considerable increased range of movement in his hip joint.

## Background

Tuberculosis (TB) is an infectious disease caused by an acid-fast bacillus called Mycobacterium tuberculosis while minority of the cases has also been attributed to M. bovis (Shembekar & Babhulkar 2002). The latest estimates made by World Health Organization included in its report are that there were 8.6 million new TB cases in the year 2012 and 1.3 million deaths resulting from TB. Furthermore, less than 1.0 million deaths were among HIV-negative cases and 0.3 million were HIV-associated TB deaths (World Health Organization). The prevalence of TB is evidently high among AIDS patients, and it’s often the first manifestation of HIV infection (Lupatkin et al. 1992; Jaber & Gleckman 1995; Moore & Rafii 2001).

Osteoarticular tuberculosis (OAT) is a rare occurrence in the field of clinical orthopedics. The tuberculous arthritis presents with high risk of deterioration of the joints involved. The delay in diagnosis which is usually witnessed in clinical practice along with inadequate therapy are two of the major problems that need to be tackled in order to improve the therapeutic results. Shembekar A et al. stated in his research that currently multi-drug anti-tuberculous chemotherapy is the mainstay treatment for OAT. And although the success rate of chemotherapy is fairly high (as much as 90%), factors such as failure to comply with optimum drug regimens or lack of an optimum regimen or irregularity in drug use and insufficient length of therapy might lead to a recurrence (Tulsi 2002). Hip joint and knee joint are the two crucial large weight bearing joints which are also the common locations for the existence of extra-axial osteoarticular TB (Al-Sayyad & Abumunaser 2013; La Fond 1958). At the present the TB of knee and hip joint accounts for 25-35% of the OAT while hip joint TB alone accounts for 15% (La Fond 1958; Babhulkar & Pande 2002).

Since the hip joint serves a wide range of function in body movements and is the most pivotal weight bearing joint in human body, the degeneration of hip joint could prove to be detrimental for the patients’ physical and emotional well-being. Furthermore the delay in diagnostic assessment culminates into long term and refractory morbidity. The therapeutic measures that are adopted are conventionally, incision and drainage or arthrodesis; however total hip arthroplasty (THA) has definite advantage over the other options with higher rate of recuperation in patients after the surgery.

## Case description

**Figure 1:**
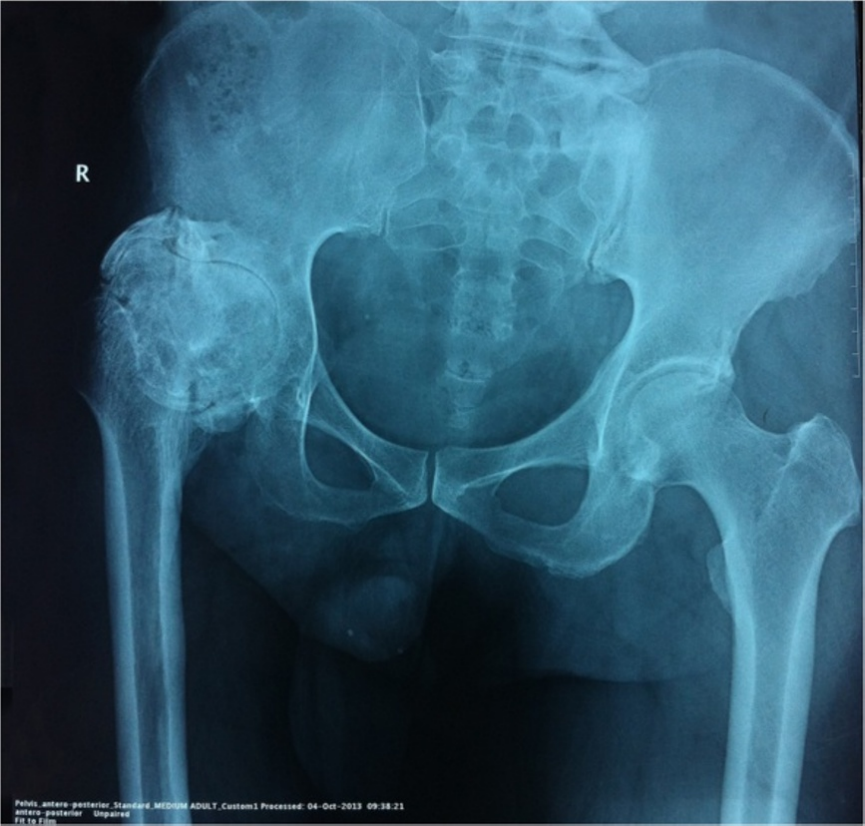
**An anterio-posterior X-ray of the hip performed preoperatively shows extraarticular spontaneous fusion of the right hip joint along with deterioration of the articular surface.**

In this case report we present a case of 54 years old male patient of Chinese descent who presented to the clinic with a complaint of mobility restriction on right lower extremity for 40 years. Upon investigation, the patient was found to have a history of right sided hip joint TB at the age of 14. The patient had increasing pain on his right lower extremity with restriction of movements especially abduction and external rotation. After seeking medical consultation, a battery of tests were conducted that led to the diagnosis. The Tuberculin purified protein derivative (PPD) test was conducted in the clinic which was positive as was demonstrated by the size of the induration formed at the site of infiltration. The patient had received anti- TB chemotherapy with isoniazid, rifampicin, pyrazinamide and ethambutol along with prophylactic antibiotics to prevent the secondary infections. The patient was not clear about the exact duration of the therapy. A progressive restriction on the right hip joint had ensued after a short time post therapy. But further intervention however was not sought since there was a certain degree of alleviation in patient’s condition. After a few years the movement restrictions had grown even more severe accompanied by pain while maneuvering the joint. On presentation, the patient had 0 degree abduction, internal and external rotation. Recent radiological findings showed right hip joint fusion along with deterioration of the articular surfaces [Figure 1]. The patient didn’t suffer from any other systemic condition.

After admittance to the Qilu Hospital of Shandong University, all the required tests were carried out that included blood work, microbiological assessment, chest and spine X-rays, CT scans, in order to exclude the persistent tuberculosis lesions in any other organ thus ultimately omitting any contraindication for the total hip arthroplasty. Sputum and urine cultures were negative. The routine blood work showed an elevated Erythrocytic Sedimentation Rate (ESR 87 mm/hr) and C-reactive protein (CRP 75 mg/L). The diagnosis was made from the patient’s history, clinical symptoms, radiological data and the histopathological reports from the surgically extricated tissue that showed granulomatous inflammatory tissue with strains of acid-fast bacilli which was confirmed as M. tuberculosis bacilli by the microbiological testing. However a prior attempt to aspirate the synovial fluid for culture was unsuccessful. The patient was put on anti-tuberculous drug regimen including isoniazid, rifampin, pyrazinamide and ethambutol for a period of 3 months before arranging the surgical procedure.

**Figure 2:**
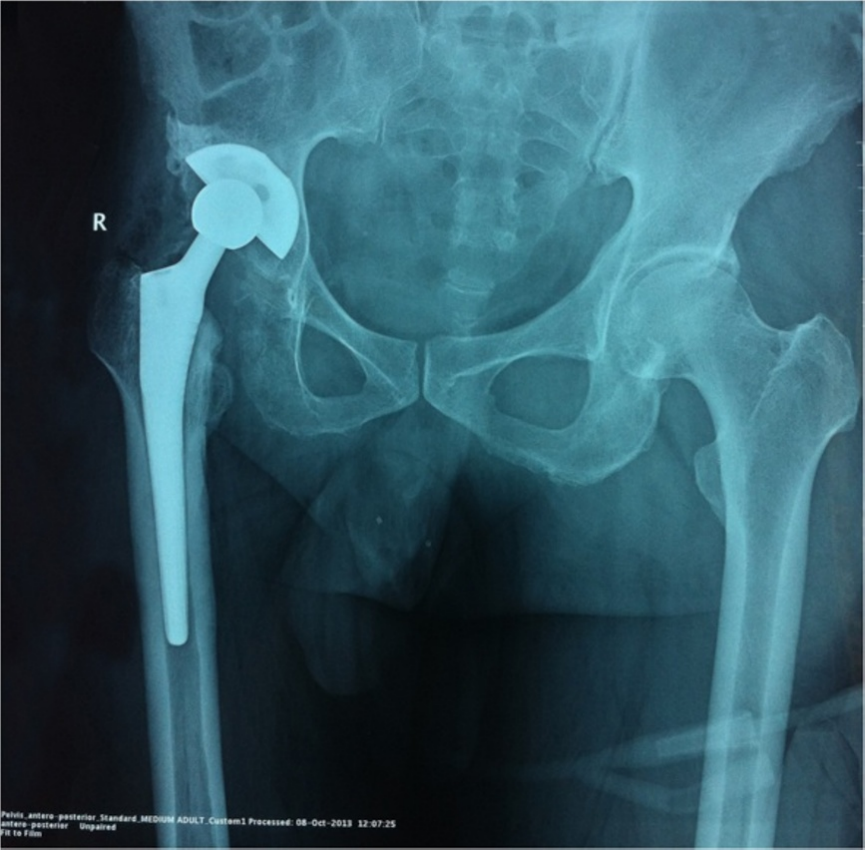
**A post-operative anterio-posterior X-ray shows placement of a metallic prosthetic joint with proper prosthetic alignment.**

**Figure 3:**
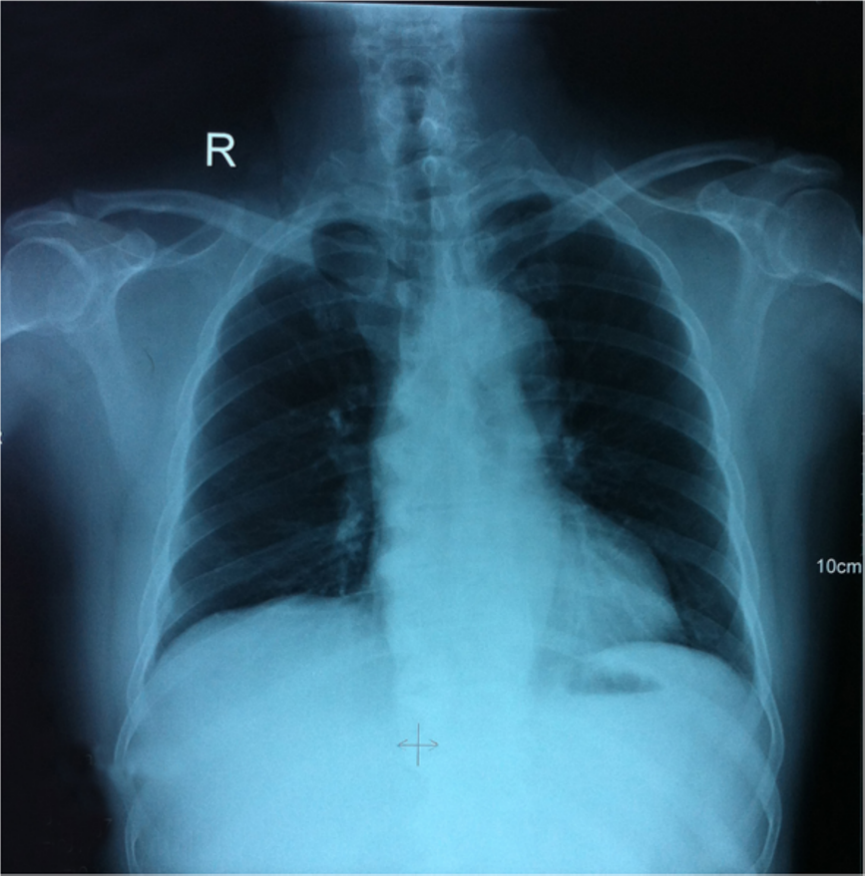
**An anterio-posterior chest X-ray was unremarkable for any tuberculotic lesions excluding the co-existence of pulmonary tuberculosis.**

Total hip arthroplasty was performed during which an excessive callus formation was observed around the femoral neck which was chiseled out from the intact bone cortex as far as possible. The inter-operative tissues were preserved for a histopathological analysis. An acetabular cup of optimal measurement was set along with a high cross linking polyethylene sleeve, then a metallic traditional femoral component was set. After reduction, the joint movements were evaluated where a flexion of 90°, abduction of 30° was attained. The X-rays taken post-surgery showed proper prosthetic alignment [Figure 2]. The postoperative recovery was unremarkable for any complication and the patient was discharged in 15 days after the surgery. Regular follow-ups were arranged to monitor the patient’s condition and he was counseled to refrain from activities that put extra strain on the weight-bearing joints. No aberrant lesions were detected in lungs or bladder or genital organs. Chest [Figure 3] and spine X-rays were devoid of any lesions thus excluding the co-existence of a pulmonary or any other extrapulmonary TB. The same regimen of anti-TB drugs as mentioned earlier was continued for one year postoperatively. Timely follow-ups were conducted for a period of 24 months during which the patient presented with a normal gait and no complaint of pain at the affected side.

### Discussion and evaluation

OATs are the most common forms of extrapulmonary TB (El Titi et al. 1987). However; the obscurity of the symptoms puts many hurdles on the path to its early diagnosis. Most patients only present with joint swelling and pain. According to a recent case study presented by Mohammed J et al. only 38% of the patients reviewed showed some systemic symptoms of tuberculosis like low-grade fever and night sweats while there was no systemic signs in the rest of the cases thus there was an average of 2 years of delay in diagnosis (Al-Sayyad & Abumunaser 2013). The delay in diagnosis in the forms of extrapulmonary TB involving sacroiliac joint might be caused by the examination traditionally carried out with patient in the supine position and also due to the physicians failure to perform the sacroiliac joint pain provocation maneuvers (Babhulkar & Pande 2002; Laslett & Williams 1994). OAT usually manifests as a monoarticular arthritic condition rather than affecting multiple joints (Grosskopf et al. 1994). Frequently Mycobacterial infection of the sacroiliac joint may present as psoas abscess and may not be diagnosed till the spontaneous drainage into the groin (Feldmann et al. 1981; Kim et al. 1999; Richter et al. 1983).

Total hip arthroplasty or total hip replacement has now emerged as one of the most common orthopedic procedures performed for debilitating lesions of the hip joint that lead to restriction of joint movement, pain and deteriorates the patient’s living standards. The therapeutic outcome of THA has been substantial in the treatment of the osteoarthritis in comparison to the conventional techniques with obvious alleviation in pain symptoms, joint movements as well as the patient’s living standards. THA is also the established method of treatment in cases of dormant TB of hips (Tulsi 2002; Kim et al. 1987). The implementation of THA in the cases of active tuberculous coxitis has been a debated concept. There are certain risks involved with THA on patients with tuberculous arthritis which can’t be undermined. There is a potential risk of reactivation of the TB infection postoperatively. Many orthopedic surgeons argue that the risks outweigh the benefits thus they suggest that anti-TB drug therapy should be started preoperatively for an optimal duration (Babhulkar & Pande 2002; Kim et al. 1987; Sidhu & Singh 2009; Xun-wu et al. 2013). In a study presented by Sidhu AS et al., the recurrent case of tuberculous arthritis was attended with a three month course of preoperative drug therapy followed by total hip replacement after which an extended duration of drug therapy was continued postoperatively (Sidhu & Singh 2009). There have been few reports which demonstrated various cases of peri-prosthetic tuberculous infection post surgery. M Kaya et al. presented a case of peri-prosthetic tuberculous infection in the patient with history of THA for osteoarthritis performed 9 years earlier (Kaya et al. 2006). Walczak P et al. presented a case report of a patient with a history of tuberculous hip in 58 years earlier who underwent THA 9 years before (Walczak et al. 2012). Thus proper prophylactic measures are crucial while undertaking this treatment method.

The limitation of our paper is its being a single patient case-report however we believe that there are very few case-reports on the recurrence of the TB of hip after a considerably long duration. Although detailed history was collected from the patient regarding compliance to the medication, it can however still be one of the fators for the recurrence of TB after a considerably long duration of time. TB is considered quiescent after 10 years of remission while some authors prolong the duration to 20 years (Kim et al. 1987; Hardinge et al. 1979). In our case the TB had relapsed after 40 years of purported remission. The therapeutic approach was extrapolated from the techniques chosen for an active TB infection of hip and there was no relapse of the disease for as long as 12 months after cessation of a year-long postoperative anti-tuberculous drug therapy although a longer follow up is still crucial.

## Conclusion

In conclusion, THA combined to optimal anti-tuberculous chemotherapy can be effective therapy for the cases of recurrence of OATs. Further study on the more beneficial therapy for such cases might lead to improvement of the standard of patient care. Also detailed researches must be conducted to the causes of relapse after long remission and the measures to be taken to prevent it.

## Consent

The patient provided written consent for the publication of this study and any accompanying images.

## Abbreviations

TB: Tuberculosis
HIV: Human immunodeficiency virus
AIDS: Acquired immune deficiency syndrome
OAT: Osteoarticular tuberculosis
THA: Total hip arthroplasty
PPD: Purified protein derivative
CRP: C- reactive protein
ESR: Erythrocyte sedimentation rate.
